# Prevalence and factors associated with long Covid in older people in the State of Paraná^*^

**DOI:** 10.1590/1980-220X-REEUSP-2024-0429en

**Published:** 2026-03-06

**Authors:** Isabela Vanessa Tavares Cordeiro Silva, Luiz Hiroshi Inoue, Franciele Aline Machado Brito, Wanessa Cristina Baccon, Giovanna Brichi Pesce, Carla Franciele Höring, Mikhael dos Santos Theodoro, Daiane Cristina Moderno Estevam Inoue, Lorhayne Silveira Dores, Natany Aparecida Batista, Patrícia Bossolani Charlo, Vanessa Denardi Antoniassi Baldissera, Débora Regina de Oliveira Moura, Maria Aparecida Salci, Lígia Carreira

**Affiliations:** 1 Universidade Estadual de Maringá, Maringá, PR, Brazil.; 2 Universidade Unicesumar, Maringá, PR, Brazil.

**Keywords:** Post-Acute COVID-19 Syndrome, Aged, Sleep Quality, Drugs of Continuous Use, COVID-19

## Abstract

**Objective:**

To analyze the factors associated with long Covid in older people 18 months after the acute phase of the disease.

**Method:**

This cross-sectional and analytical study was conducted with older individuals who had Covid-19 in 2020 and survived the disease in the state of Paraná, Brazil. Sociodemographic, lifestyle habits, health and treatment information, as well as information on signs, symptoms and sequelae of the disease were collected through telephone interviews conducted 18 months after notification or hospital discharge. For the analyses, descriptive measures, association tests, and Poisson regression models with robust variance were used.

**Results:**

Of the 345 older people who participated in the study, 224 (65%) still had some symptom or sequelae of Covid-19 18 months after the illness. Being a smoker or former smoker (PR = 2.21; 95% CI = 1.06;3.36), self-reported sleep quality (PR = 0.32; 95% CI = 0.16–0.47), and the use of continuous medication (RP = 5.24; 95% CI = 2.64–7.85; p < 0.0001) were associated with long-term Covid.

**Conclusion:**

Morbidity and polypharmacy rates are high in this population, contributing to the persistence of symptoms. In this context, it becomes essential to align health services, together with professionals, to meet the needs arising from the sequelae in older people.

## INTRODUCTION

The Covid-19 pandemic is responsible for thousands of infections and deaths caused by the worsening of the disease. Within this context, advanced age and the presence of comorbidities were considered the main risk factors for complications and deaths^(1)^.

Epidemiological data from February 2025 indicate 777 million cases of the disease with an estimated 7.1 million deaths worldwide^(2)^. In Brazil, during the same period, 39 million infections were recorded, with approximately 715,000 deaths. The state of Paraná, as of February 20, 2025, had just over 3 million cases and 47,000 deaths, of which 30,287 were people aged 60 or older^(3,4)^.

It is generally agreed in the literature that older people are more susceptible to developing the most severe forms of Covid-19. One of the factors contributing to this susceptibility is immunosenescence, a process that involves the decline of immune system function, coupled with a high burden of pre-existing diseases in this population stratum, which may contribute to the persistence of symptoms after the acute phase, a condition known as long Covid^(5)^.

Long Covid is characterized by the persistence of signs and symptoms for more than three months after the acute phase of the disease. The World Health Organization (WHO) estimates that 10 to 20% of patients who contracted Covid-19 will experience medium-to long-term sequelae. However, there is still no database with information on individuals affected by post-Covid-19 syndrome. This situation has alerted health authorities and increased the demand for specialized services^(6)^. Furthermore, long Covid can negatively impact the Basic Activities of Daily Living (ADLs) of affected individuals^(7,8)^.

Given the complexity of long Covid and its impact on physical and emotional health, especially in the older population, further studies addressing the prevalence and associated factors of this condition are still required^(7)^, given that this new demand for care and the search for services in healthcare settings demands a new model of assistance, with specialized services focused on recovery and reduction of the long-term effects of Covid-19, as well as new skills on the part of the multidisciplinary team to manage this condition, since there are still no clear guidelines regarding the treatment of long Covid^(9)^.

Therefore, considering the vulnerability of older people to long-term Covid-19, and the lack of evidence on post-Covid-19 syndrome, this study aimed to analyze the factors associated with this condition 18 months after primary infection with SARS-CoV-2.

## METHOD

### Design of Study

This is a cross-sectional, individualized, and analytical observational survey nested within a cohort entitled “Acompanhamento longitudinal de adultos e idosos que receberam alta da internação hospitalar por Covid-19 (Longitudinal follow-up of adults and older people discharged from hospitalization due to Covid-19)” (Covid-19 Paraná/UEM Cohort)^(10)^.

### Local

The study site was the State of Paraná, located in the Southern Region of Brazil. In 2021, the estimated population of the state was approximately 11.6 million inhabitants, with 1.9 million aged citizens^(11)^ and a Human Development Index (HDI) of 0.769^(12)^.

### Population

The study population consisted of older adults (60 years or older) who had Covid-19 in 2020 and survived the disease 18 months after notification or hospital discharge.

### Selection Criteria

Two databases were used to select participants: the Influenza Epidemiological Surveillance Information System (*SIVEP-Gripe*), which records cases requiring hospitalization in wards and Intensive Care Units (ICUs), and deaths, regardless of hospitalization, and the State Covid-19 Notification System (*Notifica Covid-19*), a database developed and implemented by the government of the State of Paraná, with the aim of registering cases, hospitalizations and deaths in the state itself.

Based on a preliminary analysis of the completeness of the databases containing sensitive participant information, four variables were used for the selection process: name, date of birth, sex, and mother’s name. After data processing, *SIVEP-Gripe* was used exclusively for cases requiring hospitalization, while *Notifica Covid-19* was used for mild cases, that is, those with a positive result for the disease that did not require hospitalization.

The selection criteria considered individuals aged 60 or older, residing and notified in Paraná, with a confirmed diagnosis of Covid-19 through the RT-PCR test, discharged from inpatient or ICU treatment and from outpatient care (not hospitalized) between March and December 2020, with a correctly filled-in landline or mobile phone number. Patients who died were excluded from the study.

### Sample

Initially, 1500 participants were selected from databases using proportional stratified probabilistic random sampling according to the month of notification or hospital discharge (from March to December 2020) and the macro-regional health area of residence (West, East, North and Northwest), according to the protocol described by Salci et al.^(10)^.

**Figure 1:**
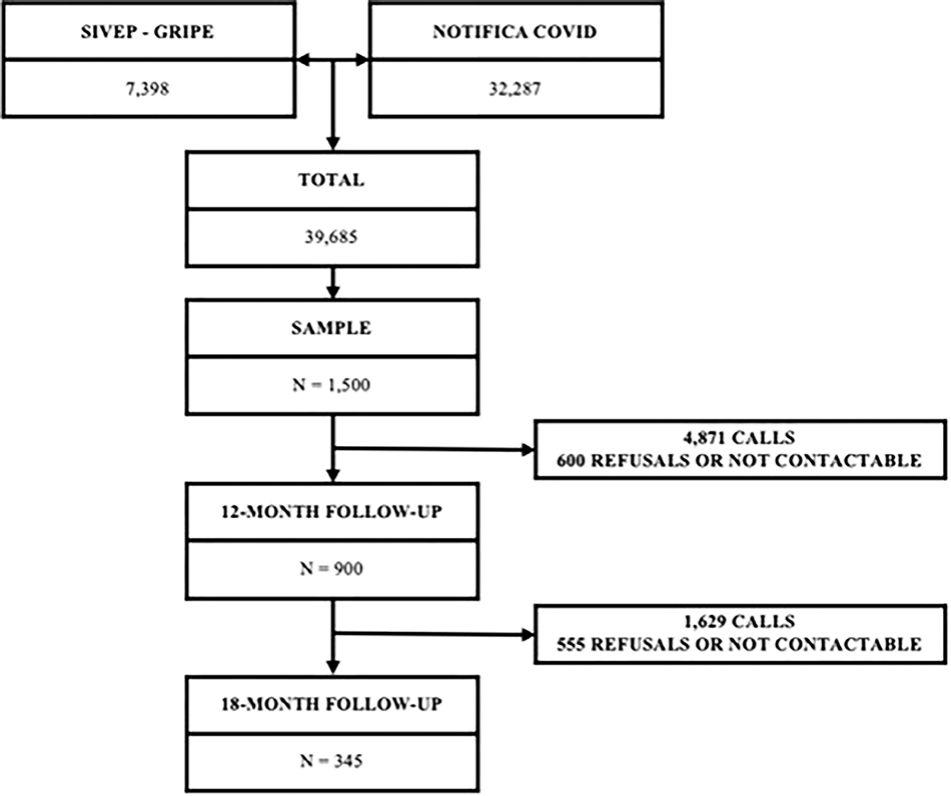
Sample selection process from databases.

Source: Prepared by the authors.

In the first telephone contact, made 12 months after notification or discharge, 900 older people agreed to participate in the research. In a second contact, 18 months after notification or hospital discharge, 345 older people effectively responded to the survey (Figure 1).

### Data Collection

Data were collected through telephone interviews conducted 18 months after notification (for mild cases) or hospital discharge (for moderate and severe cases), between November 2021 and September 2022. Individuals were invited to participate in the study by telephone by a team of nurses, health undergraduate and graduate students, who had been previously trained and qualified over a period of two months. The research objectives were explained, and verbal consent was obtained after the reading of the Informed Consent Form. In cases where the participant was unable to communicate at the time of contact, the caregiver or liable family member was allowed to answer the questions on the form. The interviews lasted an average of 30 minutes.

As a data collection instrument, a structured research form was used that included questions on sociodemographic aspects, lifestyle habits, health and treatment of Covid-19, and addressed signs and symptoms of the disease. The form was developed and validated by the cohort researchers^(10)^.

### Study Variables

The dependent variable was defined as long Covid (yes and no), characterized in this study by the presence of at least one of the self-reported signs or symptoms (listed below), persisting after 18 months from notification or hospital discharge.

The signs and symptoms evaluated were grouped according to the following systems of the human body: neurological (headache, eye pain, vision changes, loss of smell, loss of taste, speech changes, hearing changes, tinnitus, dizziness, loss of motor coordination, memory loss/decrease, tingling/numbness, fainting, fever, anxiety, and depression); respiratory (runny nose, sore throat, hoarseness, cough, phlegm production, chest pain, and dyspnea); digestive (changes in stool, changes in appetite, nausea, abdominal cramps/pain, and vomiting); endocrine (hair loss and sweating); cutaneous (spots on the body and itching); musculoskeletal (muscle/joint problems, tiredness/fatigue); and cardiovascular (edema).

The independent variables were defined in four domains: (1) sociodemographic; (2) lifestyle habits; (3) health conditions; and (4) Covid-19 treatment.

1. Sociodemographic data: sex (male and female), age (60 to 74 years and 75 years or more), race/color (white/yellow and black/indigenous), years of schooling (up to 8 and 8 or more), whether they have a partner (no and yes), whether they live alone (no and yes), and family income (up to two and more than two minimum wages);
2. Lifestyle habits: alcohol use (no and yes), smoker or former smoker (no and yes), physical activity (no and yes) and self-reported sleep quality (very poor/poor/fair and good/excellent);
3. Health conditions: has comorbidity (no and yes), continuous medication use (no and yes), sought health care in the last 18 months (no and yes), need for help/caregiver (no and yes) and presence of symptoms in the acute phase of Covid-19 (no and yes);
4. Treatment of COVID-19 in the acute phase: location (outpatient clinic, ward, and intensive care unit), use of ventilatory support (yes and no), and whether the Brazilian Public Health System (*SUS*) was used at some stage of treatment (no and yes).

Questions that participants chose not to answer were classified as “not informed”.

### Data Analysis and Treatment

Descriptive analyses of the results were performed, as well as associations between the independent variables (sociodemographic, lifestyle habits, health conditions, and Covid-19 treatment) and the outcome (presence of long-term Covid after 18 months) using Pearson’s chi-square test or Fisher’s exact test, as needed for each type of variable, at a significance level of 5%.

To identify the factors associated with persistent signs and symptoms, Poisson regression models with robust variance were applied. Associations were estimated using prevalence ratios (PR), adopting a 95% confidence interval as a measure of precision.

The final model was adjusted considering p < 0.20 in the bivariate analysis and using the “both” stepwise method for the selection of variables. The hypothesis test applied to verify the significance of the estimated coefficients was the Wald test, and the adequacy of the model was evaluated through deviance residuals analysis and randomized quantiles, and the variance inflation factor (VIF) of the independent variables. In addition, Nagelkerke’s pseudo coefficient of determination R^2^ was calculated to verify the quality of the adjustments.

The data collected in the interviews were organized into electronic spreadsheets and analyzed using the software R version 4.5.0.

### Ethical Aspects

The research was approved by the Permanent Human Research Ethics Committee, of the Universidade Estadual de Maringá/PR, under opinion no. 4.165.272/2020 and CAAE: 34787020.0.0000.0104 on July 21, 2020. Furthermore, the data obtained from *Notifica Covid* were approved by the Paraná State Health Department, through the Hospital do Trabalhador, with opinion number 4.214.589/2020 and CAAE: 34787020.0.3001.5225, on August 15, 2020.

## RESULTS

A total of 345 older people participated, with a mean age of 68 years, median of 66 years, and standard deviation of 6.6 years.

Table 1 contains the characteristics of the sample according to sociodemographic variables, lifestyle habits, health, and treatment of Covid-19.

**Table 1:** Sociodemographic, lifestyle, health, and Covid-19 treatment characteristics of older individuals 18 months after the acute phase of the disease in the State of Paraná, Brazil, 2021-2022 (n = 345) – Maringá, PR, Brazil, 2025.

Characteristics	Category	n	%
**Sociodemographic**	
Sex	Male	172	49.9
	Female	173	50.1
Age group (years)	60 to 74 years	282	81.7
	75 or more	63	18.3
Race/Color	White/Yellow	173	50.1
	Black/Indigenous	63	18.3
	Not informed	109	31.6
Years of study	Up to 8	118	34.2
	More than 8	94	27.2
	Not informed	133	38.6
Has a partner	No	83	24.1
	Yes	196	56.8
	Not informed	66	19.1
Lives alone	No	252	73.0
	Yes	51	14.8
	Not informed	42	12.2
Family income^*^	≤2 mw	60	17.4
	>2 mw	84	24.3
	Not informed	201	58.3
**Lifestyle habits**	
Consumption of alcoholic beverages	No	111	32.2
	Yes	159	46.1
	Not informed	75	21.7
Smoker or former smoker	No	148	42.9
	Yes	87	25.2
	Not informed	110	31.9
Practice of physical activity	No	69	20.0
	Yes	175	50.7
	Not informed	101	29.3
Self-reported sleep quality	Terrible/bad/average	63	18.3
	Good/excellent	242	70.1
	Not informed	40	11.6
**Health**	
Morbidity	No	123	35.7
	Yes	222	64.3
Medications for continuous use	No	165	47.8
	Yes	88	25.5
	Not informed	92	26.7
Seeking healthcare services	No	131	38.0
	Yes	137	39.7
	Not informed	77	22.3
Need for help/caregiver	No	141	40.9
	Yes	119	34.5
	Not informed	85	24.6
Symptoms in the acute phase	No	110	32.0
	Yes	235	68.0
**Treatment**			
Treatment location	Outpatient’s	148	42.9
	Ward	102	29.6
	ICU	95	27.5
Use of ventilatory support	No	172	49.9
	Yes	130	37.7
	Not informed	43	12.5
Treatment within SUS	No	2	0.6
	Yes	175	50.7
	Not informed	168	48.7

^*^The minimum wage (MW) of R$1,210.00 was used, which corresponds to the value at the beginning of the research period; SUS – Brazilian Public Health System; ICU – Intensive Care Unit.

Regarding sociodemographic information, most of the older people were between 60 and 74 years old, were female, self-identified as white/yellow, had up to eight years of schooling, lived with a partner, did not live alone, and had a family income greater than two minimum wages (Table 1).

Regarding lifestyle habits, most participants consume alcoholic beverages, are non-smokers, engage in physical activity, and consider their sleep quality to be good/excellent (Table 1).

Regarding health conditions, a large proportion have one or more comorbidities, use continuous medication, sought health care, and needed help or even a caregiver due to complications caused by Covid-19.

Regarding the treatment of the disease in the acute phase, the sample consisted mainly of people treated in outpatient clinics who did not require ventilatory support and who used the services of the *SUS* at some stage of treatment.

Among the most prevalent symptoms are memory loss/decrease, anxiety, depression, dyspnea, hair loss, muscle/joint problems, and feelings of tiredness/fatigue (Table 2).

**Table 2:** Signs and symptoms according to the human body systems of older people 18 months after the acute phase of Covid-19 in the State of Paraná, Brazil, 2021-2022 (n = 224) – Maringá, PR, Brazil, 2025.

Symptoms and signs	n	%
**Neurological**	**167**	**74.6**
Headache	29	12.9
Eye pain	27	12.1
Change in vision	38	17.0
Change in the sense of smell	31	13.8
Change in taste	29	12.9
Speech impairment	8	3.6
Hearing impairment	40	17.9
Tinnitus	34	15.2
Dizziness	51	22.8
Loss of motor coordination	38	17.0
Memory loss/reduction	123	54.9
Tingling or numbness in the body	44	19.6
Fainting	4	1.8
Fever	2	0.9
Anxiety	66	29.5
Depression	61	27.2
**Respiratory**	**107**	**47.8**
Runny nose	37	16.5
Sore throat	15	6.7
Hoarse voice	26	11.6
Cough	46	20.5
Phlegm production	24	10.7
Chest pain	17	7.6
Dyspnea	60	26.8
**Digestive system**	**56**	**25.0**
Change in stool	18	8.0
Change in appetite	37	16.5
Nausea	15	6.7
Abdominal cramps/pain	12	5.4
Vomiting	2	0.9
**Endocrine**	**68**	**30.4**
Hair loss	54	24.1
Perspiration	19	8.5
**Cutaneous**	**23**	**10.3**
Spots on the body	5	2.2
Itching all over the body	22	9.8
**Musculoskeletal**	**137**	**61.2**
Muscle/joint problems	57	25.4
Tiredness/fatigue	123	54.9
**Cardiovascular**	**17**	**7.6**
Edema	17	7.6

In the unadjusted model, the variables that were associated with long Covid (p < 0.05) were years of study, self-reported sleep quality, morbidity, continuous medication use, seeking health care, number of symptoms in the acute phase of the disease, and ICU admission (Table 3).

**Table 3:** Unadjusted model for associations of sociodemographic, lifestyle, health, treatment and long Covid-19 characteristics of older individuals 18 months after the acute phase of the disease in the State of Paraná, Brazil, 2021-2022 (n = 345) – Maringá, PR, Brazil, 2025.

Variables	Categories	Long Covid (n = 224;65%)		PR (95%CI)
		n (%)	p value	
**Sociodemographic**				
Sex	Male	104 (60.4)	0.1054	Ref.
	Female	120 (69.3)		0.88 (0.75–1.02)
Age group (years)	60 to 74 years	187 (66.3)	0.3202	Ref.
	75 or more	37 (58.7)		1.13 (0.91–1.42)
Race/Color	White/Yellow	121 (69.9)	0.7471	Ref.
	Black/Indigenous	42 (66.6)		1.06 (0.86–1.29)
Years of study	Up to 8	87 (73.7)	0.0600	Ref.
	More than 8	57 (60.6)		1.20 (0.99–1.46)
Has a partner	No	55 (66.2)	0.6473	Ref.
	Yes	137 (69.8)		0.94 (0.79–1.13)
Lives alone	No	170 (67.4)	0.9988	Ref.
	Yes	35 (68.6)		0.99 (0.80–1.21)
Family income	≤2 mw	45 (75)	0.7754	Ref.
	>2 mw	60 (71.4)		1.07 (0.88–1.30)
**Lifestyle habits**
Consumption of alcoholic beverages	No	73 (65.7)	0.4280	Ref.
	Yes	113 (71.0)		0.93 (0.79–1.10)
Smoker or former smoker	No	98 (66.2)	0.1097	Ref.
	Yes	67 (77.0)		0.87 (0.74–1.02)
Practice of physical activity	No	53 (76.8)	0.1711	Ref.
	Yes	117 (66.8)		1.14 (0.97–1.35)
Self-reported sleep quality	Terrible/bad/average	58 (92.0)	**<0.0001**	Ref.
	Good/excellent	144 (59.5)		1.54 (1.36–1.75)
**Health**				
Morbidity	No	69 (56.0)	**0.0147**	Ref.
	Yes	155 (69.8)		0.81 (0.68–0.97)
Medications for continuous use	Não	104 (63.0)	**0.0105**	Ref.
	Yes	70 (79.5)		0.80 (0.68–0.93)
Seeking healthcare services	No	68 (51.9)	**<0.0001**	Ref.
	Yes	118 (86.1)		0.61 (0.51–0.73)
Need for help/caregiver	No	99 (70.2)	0.5024	Ref.
	Yes	78 (65.5)		1.08 (0.91–1.28)
Symptoms in the acute phase	No	66 (60.0)	0.1722	Ref.
	Yes	158 (67.5)		0.89 (0.74–1.06)
**Treatment**				
Treatment location	Outpatient’s	93 (62.8)	**0.0241**	Ref.
	Ward	59 (57.8)		1.09 (0.89–1.34)
	ICU	72 (75.7)		0.83 (0.71–0.99)
Use of ventilatory support	No	111 (64.5)	0.5542	Ref.
	Yes	89 (68.4)		0.95 (0.81–1.11)
Treatment within SUS	No	1 (50.0)	0.5183^b^	Ref.
	Yes	122 (69.7)		0.71 (0.18–2.86)

ICU – Intensive Care Unit; SUS – Brazilian Public Health System; ^a^Pearson’s chi-square test;

^b^Fisher’s exact test; 95% CI – 95%confidence interval; PRu – unadjusted prevalence ratio; Ref. – reference category; Values highlighted indicate p < 0.05.

Table 4 presents the final adjusted model for the prevalence of long-term Covid in older adults according to the study’s covariates. It was observed that older people who smoked or were former smokers had a 2.21 times higher prevalence of long-term Covid compared to non-smokers. Self-reported sleep quality as good or excellent had a prevalence of 0.32 when compared to those who reported it as very poor, poor, or fair. Older people who use continuous medication showed a 5.24 times higher prevalence of long-term Covid compared to those who do not. The variables “seeking healthcare in the last 18 months” and “presence of symptoms in the acute phase of the disease” were not significant, but remained in the model due to the assumptions and goodness-of-fit tests.

**Table 4:** Final model adjusted for associations of variables lifestyle, health, and treatment of Covid-19 in older individuals 18 months after the acute phase of the disease in the State of Paraná, Brazil, 2021-2022 (n = 345) – Maringá, PR, Brazil, 2025.

Variables	Categories	PR (95% CI)	p value
Intercept	–	–	**0.0305**
Smoker or former smoker	Yes	2.21 (1.06;3.36)	**0.0270**
Self-reported sleep quality	Good/excellent	0.32 (0.16;0.47)	**0.0032**
Medications for continuous use	Yes	5.24 (2.64;7.85)	**<0.0001**
Seeking healthcare services	Yes	1.73 (0.70;2.76)	0.1516
Symptoms in the acute phase	Yes	0.23 (0.05;1.10)	0.0706

95% CI – 95% confidence interval; PR – Prevalence ratio; Highlighted values indicate p < 0.05.

The information on the treatment variables in the SUS were not included in the final model because there were not enough samples for adjustments. The use of ventilatory support showed collinearity with the treatment site, and morbidity showed collinearity with the use of continuous medication. The results of the deviance residuals tests presented p = 0.4780 and the randomized quantiles p = 0.5477, and the adjusted pseudo R^2^ result was 92.2%, the best among the models evaluated.

## DISCUSSION

The results of this analysis demonstrated that older people, being more vulnerable to Covid-19, became more susceptible to long Covid. Furthermore, they showed that those who rate their sleep quality as good or excellent are less likely to develop persistent symptoms. In turn, those who sought healthcare and use continuous medication are more likely to suffer from long Covid-19 symptoms 18 months after the primary SARS-CoV-2 infection. Corroborating these findings, the literature also indicates that individuals over 60 years of age are more likely to develop long-term complications from Covid-19^(5)^.

One of these changes relates to sleep quality, with several studies stating that individuals experience changes in their sleep patterns after contracting the coronavirus. A study conducted in 56 countries, where 3,726 participants were followed for seven months, identified sleep disorders in 78.6% of the sample, with 60% reporting insomnia, 41% night sweats, 36% waking up during sleep, and 10% sleep apnea^(13)^.

Older adults who experience sleep disorders are more predisposed to developing neuropsychiatric changes, such as anxiety, depression, fatigue, decreased concentration, cognitive dysfunction, and paresthesia. Therefore, having good sleep quality reduces the occurrence of these events that are present in most individuals with long-term Covid^(14)^.

In Hubei province, China, the epicenter of the pandemic, it was found that after the acute phase of the disease, 17.7% of older people presented alterations in their sleep patterns^(15)^. Similar results from a prospective cohort study conducted in Hong Kong with the same population found that 14.0% reported sleep disturbances; insomnia and poor sleep quality were the most prevalent symptoms^(16)^. In this study, most participants reported having good to excellent sleep quality, which was identified as a protective factor against long-term Covid.

It is important to highlight that older people experience a negative response to the coronavirus, presenting sequelae and complications that affect their motor skills and cognitive functions after the acute phase of the disease, a condition known in the long term as long Covid^(17)^.

Another finding of the study, which has been described in other works, is the use of continuous medication as a predictor of long Covid. A survey conducted in four Chinese cities, a study of 2,712 individuals with persistent symptoms, identified that 7.3% of the sample reported continuous medication use before contracting Covid-19, making them susceptible^(18)^. Other studies indicate that, depending on individual needs, long-term effects of Covid are treated with daily medication, including anxiolytics, antidepressants, and allergy medications, aiming to minimize post-Covid effects^(19)^.

According to data from a cohort study conducted in the United States, one year after contracting Covid-19 there was an increase in medication prescriptions following the acute phase of the illness, including vitamins and anticoagulants^(20)^. Corroborating these findings, another study showed that the use of medications used to treat comorbidities, mostly found in older people, are associated with long Covid^(21)^.

A cohort study conducted in Iran followed individuals for three months after a Covid-19 diagnosis to identify factors associated with long Covid. Clinical assessments and sample collection were performed, and the researchers observed psychological changes and the persistence of signs and symptoms in the participants. The study sample consisted of 900 patients who were hospitalized in early 2020 and subsequently discharged; of these, 350 underwent health follow-up after the acute phase of the disease. One can assume how necessary it is to seek healthcare services to treat the after-effects of the illness^(22)^.

Long-term care following Covid-19 is essential for recovery. Effective communication between in-hospital teams and primary care services is required, to ensure the availability of information on patients’ health conditions and the supply of specialized services to meet their needs^(23)^. It is known that individuals who contracted the coronavirus were classified as having mild, moderate, and severe cases, with most older people presenting severe cases; therefore, these individuals require more frequent follow-up^(24)^.

Older adults have a higher overall burden of chronic diseases, which the literature highlights as a contributing factor to the persistence of symptoms and complications after the acute phase of the disease. Therefore, there is a need for specialized services and trained professionals to provide healthcare and treatment tailored to the specific needs of each individual. In this sense, attention to the older people is essential to reduce harm and improve their quality of life^(25)^.

Notably, even with the end of the emergency phase of the Covid-19 pandemic, new challenges and care demands have emerged, highlighting the need for care models focused on the older individuals^(13,26)^.

In this scenario, demands related to long Covid are mostly being directed to Primary Health Care (PHC), which provides low-complexity treatments. Conversely, several challenges arise, including a lack of health information after discharge, delays in obtaining care from PHC, and a lack of knowledge about this condition, which demands training focused on the topic of long Covid and its implications for public health^(27)^.

One limitation of the study is the number of losses during collection. There was no agreement for interviews in numerous contacts made due to participants’ apprehension regarding malicious phone calls. Another limitation relates to recall bias, which can occur in retrospective interviews. However, another reason could be the older person’s lack of understanding of the subject matter, especially since it is a remote format, in addition to the complexity of the standardized instrument, which may hinder its comprehension by the respondents.

Considering the difficulties older participants faced in responding to the data collection instrument, the authors suggest that future studies should adopt approaches that are more accessible and adapted to this population. This may include language simplification, use of face-to-face or assisted interviews, and strategies that facilitate understanding and expression of the experiences of older adults. Furthermore, they recommend using resources appropriate for this age group, ensuring an analysis tailored to the characteristics of long Covid in this group.

Therefore, it is important to emphasize the need for new studies related to long Covid, especially those with longer periods after diagnosis, so as that the associated factors and the persistence of signs and symptoms in this age group are based on more consistent evidence in the literature.

In this respect, the findings of this research contribute to enabling health managers to develop and implement future public policies based on the literature. This way, assertive actions can be carried out aimed at caring for older people diagnosed with long Covid, focusing on health recovery and prevention of complications.

## CONCLUSION

In this study, being a current or former smoker and continuous use of medication were associated with long Covid. Self-reported good or excellent sleep quality was associated with long Covid as a protective factor.

Considering the high burden of comorbidities, the frequent use of multiple medications, and the vulnerability of the older people to infectious diseases, it is essential that health services and professionals are prepared to meet the needs of those living with the sequelae of SARS-CoV-2.

The findings of this study contribute to the understanding of long Covid in older adults, expanding scientific knowledge and highlighting the need to improve strategies for this condition.

It is expected that this data will drive progress in the study of long Covid in the older individuals, providing essential information for the creation of health practices and policies that promote recovery and improve this population’s quality of life.

## Data Availability

The entire dataset supporting the results of this study was published in the article itself.
