# Supplementary material for: Prevalence and factors associated with long Covid in older people in the State of Paraná
Source: Rev Esc Enferm USP. 2026 Mar 6;60:e20240429. doi: 10.1590/1980-220X-REEUSP-2024-0429en (PMC12975296; doi:10.1590/1980-220X-REEUSP-2024-0429en)
Supplement: Translation

# Translation: Prevalência e fatores associados à Covid longa em pessoas idosas no Estado do Paraná^*^

**DOI:** 10.1590/1980-220X-REEUSP-2024-0429pt

**Authors:** Isabela Vanessa Tavares Cordeiro Silva, Luiz Hiroshi Inoue, Franciele Aline Machado Brito, Wanessa Cristina Baccon, Giovanna Brichi Pesce, Carla Franciele Höring, Mikhael dos Santos Theodoro, Daiane Cristina Moderno Estevam Inoue, Lorhayne Silveira Dores, Natany Aparecida Batista, Patrícia Bossolani Charlo, Vanessa Denardi Antoniassi Baldissera, Débora Regina de Oliveira Moura, Maria Aparecida Salci, Lígia Carreira

**Affiliations:** 1 Universidade Estadual de Maringá, Maringá, PR, Brasil.; 2 Universidade Unicesumar, Maringá, PR, Brasil.

**Keywords:** Síndrome de Pós-COVID-19 Aguda, Idoso, Qualidade do Sono, Medicamentos de Uso Contínuo, COVID-19

## Abstract

**Objetivo:**

Analisar os fatores associados à Covid longa em pessoas idosas após 18 meses da fase aguda da doença.

**Método:**

Estudo transversal e analítico realizado com idosos que tiveram Covid-19 no ano de 2020 e sobreviveram à doença no estado do Paraná, Brasil. Informações sociodemográficas, de hábitos de vida, de saúde e de tratamento, bem como sobre sinais, sintomas e sequelas da doença foram coletados por meio de entrevistas telefônicas realizadas 18 meses após a notificação ou a alta hospitalar. Para as análises, foram utilizadas medidas descritivas, testes de associação e modelos de regressão de Poisson com variância robusta.

**Resultados:**

Das 345 pessoas idosas que participaram do estudo, 224 (65%) ainda apresentavam algum sintoma ou sequela da Covid-19 após 18 meses da doença. Foram associados à Covid longa ser tabagista ou ex-tabagista (RP = 2,21; IC 95% = 1,06;3,36), a qualidade do sono autorreferida (RP = 0,32; IC 95% = 0,16–0,47) e o uso de medicação contínua (RP = 5,24; IC 95%: 2,64–7,85; p < 0,0001).

**Conclusão:**

As taxas de morbidade e polifarmácia nessa população são altas, contribuindo para a permanência de sintomas. Neste contexto, torna-se fundamental o alinhamento dos serviços de saúde, em conjunto com os profissionais, a fim de suprir as necessidades provenientes das sequelas nas pessoas idosas.

## INTRODUÇÃO

A pandemia da Covid-19 é responsável por milhares de infecções e mortes ocasionadas pelo agravamento da doença. E dentro desse contexto, a idade avançada e a presença de comorbidades foram consideradas os principais fatores de risco para complicações e mortes^(1)^.

Dados epidemiológicos de fevereiro de 2025 apontam 777 milhões de casos da doença com uma estimativa de 7,1 milhões de óbitos em todo o mundo^(2)^. No Brasil, no mesmo período foram registrados 39 milhões de infectados, com aproximadamente 715 mil mortes. O Estado do Paraná, até o dia 20 de fevereiro de 2025 apresentava pouco mais de 3 milhões de casos e 47 mil óbitos, dentre estes, 30.287 são de pessoas com 60 anos ou mais^(3,4)^.

É consenso na literatura que pessoas idosas são mais suscetíveis a desenvolver as formas mais graves da Covid-19. Um dos fatores que contribui para essa susceptibilidade é a imunossenescência, processo que consiste no declínio das funções do sistema imune, somado à alta carga de doenças pré-existentes nesse estrato populacional, e pode contribuir para a permanência dos sintomas após a fase aguda, uma condição conhecida por Covid longa^(5)^.

A Covid longa é caracterizada pela permanência de sinais e sintomas por mais de três meses após a fase aguda da doença. A Organização Mundial da Saúde (OMS) estima que 10 a 20% dos pacientes que contraíram a Covid-19 irão apresentar sequelas em médio e longo prazo. No entanto, ainda não há uma base de dados com informações sobre os indivíduos que estão acometidos pela síndrome pós-Covid-19. Tal condição tem alertado as autoridades em saúde e aumentado a demanda por serviços especializados^(6)^. Além disso, a Covid longa pode impactar negativamente as Atividades Básicas de Vida Diária (ABVD) dos indivíduos acometidos^(7,8)^.

Frente à complexidade da Covid longa e o impacto que ela traz à saúde física e emocional, especialmente na população idosa, ainda são necessários estudos que abordem a prevalência e os fatores associados a essa condição^(7)^, visto que essa nova demanda de cuidados e busca por atendimento nos serviços de saúde configura a necessidade de um novo formato de assistência, com serviços especializados voltados à recuperação e à redução das sequelas pós Covid-19, assim como novas competências por parte da equipe multiprofissional para manejar essa condição, já que ainda não existem diretrizes claras quanto ao tratamento da Covid longa^(9)^.

Dessa forma, considerando a vulnerabilidade das pessoas idosas frente à Covid longa, e a falta de evidências sobre a síndrome pós-Covid-19, esse estudo propôs analisar os fatores associados a essa condição após 18 meses da infecção primária pelo SARS-CoV-2.

## MÉTODO

### Desenho do Estudo

Trata-se de um inquérito observacional transversal, individuado e analítico aninhado a uma coorte intitulada “Acompanhamento longitudinal de adultos e idosos que receberam alta da internação hospitalar por Covid-19” (Coorte Covid-19 Paraná/UEM)^(10)^.

### Local

O local de estudo foi o Estado do Paraná, localizado na Região Sul do Brasil. Em 2021, a população estimada no Estado era de aproximadamente 11,6 milhões de habitantes, com 1,9 milhões de idosos^(11)^ e um Índice de Desenvolvimento Humano (IDH) de 0,769^(12)^.

### População

A população do estudo foi composta por idosos (60 anos ou mais) que tiveram Covid-19 no ano de 2020 e sobreviveram à doença 18 meses após a notificação ou alta hospitalar.

### Critérios de Seleção

Foram utilizadas duas bases de dados para a seleção dos participantes: o Sistema de Informação da Vigilância Epidemiológica da Gripe (SIVEP-Gripe), onde se registram os casos que necessitam de internamento em enfermarias e Unidades de Terapia Intensiva (UTI), e os óbitos, independentemente de internação, e o Sistema Estadual Notifica Covid-19 (Notifica Covid-19), uma base de dados elaborada e implementada pelo governo do Estado do Paraná, com o intuito de registrar os casos, internamentos e óbitos do próprio estado.

A partir de uma análise prévia de completude dos bancos de dados contendo as informações sensíveis dos participantes, quatro variáveis foram utilizadas para o processo de seleção: nome, data de nascimento, sexo e nome da mãe. Após o tratamento dos dados, o SIVEP-Gripe foi utilizado exclusivamente para os casos que necessitaram de internação, enquanto o Notifica Covid-19 foi empregado para os casos leves, ou seja, aqueles com resultado positivo para a doença que não precisaram de internação.

Como critérios de seleção foram considerados indivíduos com 60 anos ou mais, residentes e notificados no Paraná, com diagnóstico confirmado de Covid-19 por meio do teste RT-PCR, egressos de tratamento em enfermaria ou UTI e de atendimentos ambulatoriais (não hospitalizados) no período de março a dezembro de 2020, com número de telefone fixo ou celular preenchido corretamente. Foram excluídos do estudo os pacientes que evoluíram para óbito.

### Amostra

Inicialmente, foram selecionados 1500 participantes a partir de bases de dados utilizando amostragem aleatória probabilística estratificada proporcional segundo o mês de notificação ou alta hospitalar (de março a dezembro de 2020) e a macrorregional de saúde de residência (Oeste, Leste, Norte e Noroeste), conforme o protocolo descrito por Salci et al.^(10)^.

No primeiro contato telefônico, realizado 12 meses após a notificação ou alta, 900 pessoas idosas concordaram em participar da pesquisa. Em um segundo contato, 18 meses após a notificação ou alta hospitalar, 345 idosos efetivamente responderam à pesquisa (Figura 1).

**Figura 1:** Processo de seleção da amostra a partir das bases de dados.

Fonte: Elaborado pelos autores.

### Coleta de Dados

Os dados foram coletados por meio de entrevistas telefônicas realizadas 18 meses após a notificação (para os casos leves) ou a alta hospitalar (para casos moderados e graves), período entre novembro de 2021 e setembro de 2022. Os indivíduos foram convidados a participar do estudo por telefone por uma equipe de enfermeiros(as), estudantes de graduação e pósgraduação da área da saúde, que foi treinada e capacitada previamente durante dois meses. Foram esclarecidos os objetivos da pesquisa e a anuência verbal era realizada após a leitura do Termo de Consentimento Livre e Esclarecido (TCLE). Em casos de incapacidade de comunicação do participante no momento do contato, foi permitido que o cuidador ou o familiar responsável respondesse às questões do formulário. As entrevistas tiveram duração média de 30 minutos.

Como instrumento de coleta dos dados, foi utilizado um formulário estruturado de pesquisa que compreendia questões sociodemográficas, de hábitos de vida, de saúde e de tratamento da Covid-19, e abordava sinais e sintomas da doença. O formulário foi elaborado e validado pelos pesquisadores da coorte^(10)^.

### Variáveis do Estudo

Definiu-se como variável dependente a Covid longa (sim e não), caracterizada neste estudo pela presença de pelo menos um dos sinais ou sintomas autorreferidos (listados a seguir), persistentes após 18 meses da notificação ou da alta hospitalar.

Os sinais e sintomas avaliados foram agrupados de acordo com os seguintes sistemas do corpo humano: neurológico (cefaleia, dor nos olhos, alteração na visão, alteração no olfato, alteração no paladar, alteração na fala, alteração na audição, zumbido no ouvido, tontura, perda de coordenação motora, perda/diminuição da memória, formigamento/dormência, desmaio, febre, ansiedade e depressão); respiratório (coriza, dor na garganta, voz rouca, tosse, produção de catarro, dor torácica e dispneia); digestório (alteração nas fezes, alteração do apetite, náusea, cólica/dor abdominal e vômito); endócrino (queda de cabelo e transpiração); cutâneo (manchas pelo corpo e prurido no corpo); musculoesquelético (problemas nos músculos/articulações, cansaço/fadiga); e cardiovascular (edema).

As variáveis independentes foram definidas em quatro domínios: (1) sociodemográficas; (2) hábitos de vida; (3) condições de saúde; e (4) tratamento da Covid-19.

1. Sociodemográficas: sexo (masculino e feminino), idade (60 a 74 anos e 75 anos ou mais), raça/cor (branca/amarela e negra/indígena), anos de estudo (até 8 e 8 ou mais), se possui companheiro(a) (não e sim), se reside sozinho(a) (não e sim) e renda familiar (até dois e mais de dois salários mínimos);
2. Hábitos de vida: uso de bebida alcoólica (não e sim), tabagista ou ex-tabagista (não e sim), prática de atividade física (não e sim) e qualidade do sono autorreferida (péssimo/ruim/regular e bom/excelente);
3. Condições de saúde: possui morbidade (não e sim), medicamentos de uso contínuo (não e sim), busca por atendimento de saúde nos últimos 18 meses (não e sim), necessidade de ajuda/cuidador (a) (não e sim) e presença de sintomas na fase aguda da Covid-19 (não e sim);
4. Tratamento da Covid-19 na fase aguda: local (ambulatório, enfermaria e unidade de terapia intensiva), uso de suporte ventilatório (não e sim) e se utilizou o Sistema Único de Saúde (SUS) em alguma fase do tratamento (não e sim).

Questões que os participantes optaram por não responder foram classificadas como “não informado”.

### Análise e Tratamento dos Dados

Foram realizadas análises descritivas dos resultados, bem como associações das variáveis independentes (sociode-mográficas, hábitos de vida, condições de saúde e tratamento da Covid-19) com o desfecho (presença de Covid longa após 18 meses) por meio do teste qui-quadrado de Pearson ou exato de Fisher, conforme necessário para cada tipo de variável, ao nível de significância de 5%.

Para identificar os fatores associados aos sinais e sintomas persistentes, aplicaram-se modelos de regressão de Poisson com variância robusta. As associações foram estimadas por meio da razão de prevalências (RP), adotando um intervalo de confiança de 95% como medida de precisão.

O modelo final foi ajustado considerando p < 0,20 na análise bivariada e utilizando o *método stepwise both* para a seleção das variáveis. O teste de hipóteses aplicado para verificar a significância dos coeficientes estimados foi o teste de Wald e a adequação do modelo foi avaliada por meio da análise dos resíduos *deviance* e quantílicos aleatorizados, e do fator de inflação da variância (VIF) das variáveis independentes. Além disso, foi calculado o pseudo coeficiente de determinação R^2^ de Nagelkerke para verificar a qualidade dos ajustes.

Os dados coletados nas entrevistas foram organizados em planilhas eletrônicas e analisados por meio do *software* R versão 4.5.0.

### Aspectos Éticos

A pesquisa foi aprovada pelo Comitê Permanente de Ética em Pesquisa Envolvendo Seres Humanos, da Universidade Estadual de Maringá/PR, sob o parecer n. 4.165.272/2020 e CAAE: 34787020.0.0000.0104 em 21 de julho de 2020. Recebeu, ainda, para os dados obtidos do Notifica Covid, a aprovação da Secretaria de Estado da Saúde do Paraná, por meio do Hospital do Trabalhador, obtendo o parecer n. 4.214.589/2020 e CAAE: 34787020.0.3001.5225, em 15 de agosto de 2020.

## RESULTADOS

Participaram do estudo 345 pessoas idosas, com média de idade de 68 anos, mediana de 66 anos e desvio padrão de 6,6 anos.

A Tabela 1 contém as características da amostra segundo as variáveis sociodemográficas, hábitos de vida, de saúde e tratamento da Covid-19.

**Tabela 1:** Características sociodemográficas, de hábitos de vida, de saúde e de tratamento da Covid-19 de idosos 18 meses após a fase aguda da doença no Estado do Paraná, Brasil, 2021-2022 (n= 345) – Maringá, PR, Brasil, 2025.

Características	Categoria	n	%
**Sociodemográficas**			
Sexo	Masculino	172	49,9
	Feminino	173	50,1
Faixa etária (anos)	60 a 74 anos	282	81,7
	75 ou mais	63	18,3
Raça/Cor	Branca/Amarela	173	50,1
	Negra/Indígena	63	18,3
	Não informado	109	31,6
Anos de estudo	Até 8	118	34,2
	Mais que 8	94	27,2
	Não informado	133	38,6
Possui companheiro(a)	Não	83	24,1
	Sim	196	56,8
	Não informado	66	19,1
Reside sozinho	Não	252	73,0
	Sim	51	14,8
	Não informado	42	12,2
Renda familiar^*^	≤2 s.m.	60	17,4
	>2 s.m.	84	24,3
	Não informado	201	58,3
**Hábitos de vida**			
Consumo de bebida alcoólica	Não	111	32,2
	Sim	159	46,1
	Não informado	75	21,7
Tabagista ou ex-tabagista	Não	148	42,9
	Sim	87	25,2
	Não informado	110	31,9
Prática de atividade física	Não	69	20,0
	Sim	175	50,7
	Não informado	101	29,3
Qualidade do sono autorreferida	Péssima/ruim/regular	63	18,3
	Boa/excelente	242	70,1
	Não informado	40	11,6
**Saúde**			
Morbidade	Não	123	35,7
	Sim	222	64,3
Medicamentos de uso	Não	165	47,8
contínuo	Sim	88	255
	Não informado	92	26,7
Busca por atendimento de saúde	Não	131	38,0
	Sim	137	39,7
	Não informado	77	22,3
Necessidade de ajuda/cuidador(a)	Não	141	40,9
	Sim	119	34,5
	Não informado	85	24,6
Sintomas na fase aguda	Não	110	32,0
	Sim	235	68,0
**Tratamento**			
Local de tratamento	Ambulatório	148	42,9
	Enfermaria	102	29,6
	UTI	95	27,5
Uso de suporte ventilatório	Não	172	49,9
	Sim	130	37,7
	Não informado	43	12,5
Tratamento no SUS	Não	2	0,6
	Sim	175	50,7
	Não informado	168	48,7

*Foi utilizado o salário mínimo (s.m.) de R$1.210,00, que corresponde ao valor no período de início da pesquisa; SUS – Sistema Único de Saúde; UTI-Unidade de – Terapia Intensiva.

Para as informações sociodemográficas, a maior parte dos idosos tinha idade entre 60 a 74 anos, era do sexo feminino, a raça/cor autorreferida era branca/amarela, tinha até oito anos de estudo, vivia com companheiro (a), não residia sozinho (a) e apresentava renda familiar maior que dois salários mínimos (Tabela 1).

No tocante aos hábitos de vida, a maioria dos participantes faz uso de bebidas alcoólicas, não é tabagistas, pratica atividade física e considera a qualidade do sono como boa/excelente (Tabela 1).

No que se refere às condições de saúde, grande parte possui uma ou mais morbidades, faz uso de medicação contínua, buscou atendimento de saúde e necessitou de ajuda ou até mesmo de cuidador devido às complicações causadas pela Covid-19.

Em relação ao tratamento da doença na fase aguda, a amostra foi composta majoritariamente por pessoas atendidas em ambulatório, que não necessitaram de suporte ventilatório e que utilizaram os serviços do SUS em alguma fase do tratamento.

Entre os mais prevalentes, destacam-se a perda/diminuição da memória, ansiedade, depressão, dispneia, queda de cabelo, problemas nos músculos/articulações e sensação de cansaço/fadiga (Tabela 2).

**Tabela 2:** Sinais e sintomas segundo os sistemas do corpo humano de idosos 18 meses após a fase aguda da Covid-19 no Estado do Paraná, Brasil, 2021-2022 (n = 224) – Maringá, PR, Brasil, 2025.

Sinais e sintomas	n	%
**Neurológico**	**167**	**74,6**
Cefaleia	29	12,9
Dor nos olhos	27	12,1
Alteração na visão	38	17,0
Alteração no olfato	31	13,8
Alteração no paladar	29	12,9
Alteração na fala	8	3,6
Alteração na audição	40	17,9
Zumbido no ouvido	34	15,2
Tontura	51	22,8
Perda de coordenação motora	38	17,0
Perda/diminuição da memória	123	54,9
Formigamento ou dormência no corpo	44	19,6
Desmaio	4	1,8
Febre	2	0,9
Ansiedade	66	29,5
Depressão	61	27,2
**Respiratório**	**107**	**47,8**
Coriza	37	16,5
Dor na garganta	15	6,7
Voz rouca	26	11,6
Tosse	46	20,5
Produção de catarro	24	10,7
Dor torácica	17	7,6
Dispneia	60	26,8
**Digestório**	**56**	**25,0**
Alteração nas fezes	18	8,0
Alteração no apetite	37	16,5
Náusea	15	6,7
Cólica/dor abdominal	12	5,4
Vômito	2	0,9
**Endócrino**	**68**	**30,4**
Queda de cabelo	54	24,1
Transpiração	19	8,5
**Cutâneo**	**23**	**10,3**
Manchas pelo corpo	5	2,2
Prurido no corpo	22	9,8
**Musculoesquelético**	**137**	**61,2**
Problemas nos músculos/articulações	57	25,4
Cansaço/fadiga	123	54,9
**Cardiovascular**	**17**	**7,6**
Edema	17	7,6

No modelo bruto, as variáveis que se associaram à Covid longa (p < 0,05) foram anos de estudo, qualidade do sono autorreferida, morbidade, medicação de uso contínuo, busca por atendimento de saúde, número de sintomas na fase aguda da doença e internação em UTI (Tabela 3).

**Tabela 3:** Modelo bruto para associações das características sociodemográficas, hábitos de vida, de saúde, tratamento e Covid longa em idosos 18 meses após a fase aguda da doença no Estado do Paraná, Brasil, 2021-2022 (n = 345) – Maringá, PR, Brasil, 2025.

Variáveis	Categorias	Covid longa (n = 224;65%)		RP (IC95%)
		n (%)	p-valor	
**Sociodemográficas**			
Sexo	Masculino	104 (60,4)	0,1054	Ref.
	Feminino	120 (69,3)		0,88 (0,75–1,02)
Faixa etária (anos)	60 a 74 anos	187 (66,3)	0,3202	Ref.
	75 ou mais	37 (58,7)		1,13 (0,91–1,42)
Raça/Cor	Branca/Amarela	121 (69,9)	0,7471	Ref.
	Negra/Indígena	42 (66,6)		1,06 (0,86–1,29)
Anos de estudo	Até 8	87 (73,7)	0,0600	Ref.
	Mais que 8	57 (60,6)		1,20 (0,99–1,46)
Possui companheiro(a)	Não	55 (66,2)	0,6473	Ref.
	Sim	137 (69,8)		0,94 (0,79–1,13)
Reside sozinho	Não	170 (67,4)	0,9988	Ref.
	Sim	35 (68,6)		0,99 (0,80–1,21)
Renda familiar	≤2 s.m.	45 (75)	0,7754	Ref.
	>2 s.m.	60 (71,4)		1,07 (0,88–1,30)
**Hábitos de vida**			
Consumo de bebida alcoólica	Não	73 (65,7)	0,4280	Ref.
	Sim	113 (71,0)		0,93 (0,79–1,10)
Tabagista ou ex-tabagista	Não	98 (66,2)	0,1097	Ref.
	Sim	67 (77,0)		0,87 (0,74–1,02)
Prática de atividade física	Não	53 (76,8)	0,1711	Ref.
	Sim	117 (66,8)		1,14 (0,97–1,35)
Qualidade do sono autorreferida	Péssima/ruim/regular	58 (92,0)	**<0,0001**	Ref.
	Boa/excelente	144 (59,5)		1,54 (1,36–1,75)
**Saúde**			
Morbidade	Não	69 (56,0)	**0,0147**	Ref.
	Sim	155 (69,8)		0,81 (0,68–0,97)
Medicamentos de uso contínuo	Não	104 (63,0)	**0,0105**	Ref.
	Sim	70 (79,5)		0,80 (0,68–0,93)
Busca por atendimento de saúde	Não	68 (51,9)	**<0,0001**	Ref.
	Sim	118 (86,1)		0,61 (0,51–0,73)
Necessidade de ajuda/cuidador(a)	Não	99 (70,2)	0,5024	Ref.
	Sim	78 (65,5)		1,08 (0,91–1,28)
Sintomas na fase aguda	Não	66 (60,0)	0,1722	Ref.
	Sim	158 (67,5)		0,89 (0,74–1,06)
**Tratamento**			
Local de tratamento	Ambulatorial	93 (62,8)	**0,0241**	Ref.
	Enfermaria	59 (57,8)		1,09 (0,89–1,34)
	UTI	72 (75,7)		0,83 (0,71–0,99)
Uso de suporte ventilatório	Não	111 (64,5)	0,5542	Ref.
	Sim	89 (68,4)		0,95 (0,81–1,11)
Tratamento no SUS	Não	1 (50,0)	0,5183^b^	Ref.
	Sim	122 (69,7)		0,71 (0,18–2,86)

UTI – Unidade de Terapia Intensiva;SUS – Sistema Único de Saúde;^a^Teste Qui-quadrado de Pearson

^b^Teste exato de Fisher;IC 95% – Intervalo de confiança de 95%;RPb – Razão de prevalências bruta; Ref. – categoria de referência;Valores em destaque indicam p < 0,05.

A Tabela 4 apresenta o modelo final ajustado para a prevalência da Covid longa em pessoas idosas segundo as covariáveis do estudo. Observou-se que pessoas idosas tabagistas ou ex-tabagistas apresentaram prevalência de Covid longa 2,21 vezes maior quando comparadas às não tabagistas. A qualidade do sono autorreferida como boa ou excelente apresentou prevalência de 0,32 quando comparada àqueles que a referiram como péssima, ruim ou regular. Pessoas idosas que fazem uso de medicação contínua apresentaram prevalência de Covid longa 5,24 vezes maior quando comparadas às que não utilizam. As variáveis busca por atendimento de saúde nos últimos 18 meses e presença de sintomas na fase aguda da doença não foram significativas, mas permaneceram no modelo devido aos pressupostos e testes de adequações do ajuste.

**Tabela 4:** Modelo final ajustado para associações das variáveis de hábitos de vida, de saúde e de tratamento da Covid-19 em idosos 18 meses após a fase aguda no Estado do Paraná, Brasil, 2021-2022 (n = 345) – Maringá, PR, Brasil, 2025.

Variáveis	Categorias	RP (IC 95%)	p-valor
Intercepto	–	–	**0,0305**
Tabagista ou ex-tabagista	Sim	2,21 (1,06;3,36)	**0,0270**
Qualidade do sono autorreferida	Boa/excelente	0,32 (0,16;0,47)	**0,0032**
Medicamentos de uso contínuo	Sim	5,24 (2,64;7,85)	**<0,0001**
Busca por atendimento de saúde	Sim	1,73 (0,70;2,76)	0,1516
Sintomas na fase aguda	Sim	0,23 (0,05;1,10)	0,0706

IC 95% – Intervalo de confiança de 95%; RP – Razão de prevalências; Valores em destaque indicam p < 0,05.

As informações das variáveis tratamento no SUS não foram imputadas no modelo final por não conterem amostras suficientes para os ajustes. O uso de suporte ventilatório apresentou colinearidade com o local de tratamento e a morbidade colinearidade com o uso de medicação contínua. Os resultados dos testes dos resíduos *deviance* apresentaram p = 0,4780 e dos quantílicos aleatorizados p = 0,5477, e o resultado do pseudo R^2^ ajustado foi de 92,2%, o melhor entre os modelos avaliados.

## DISCUSSÃO

Os resultados desta análise demonstraram que pessoas idosas, por serem mais vulneráveis a Covid-19, tornaram-semais suscetíveis a Covid longa. Além disso, evidenciaram que aqueles que referem a qualidade do sono como boa e excelente têm menos chance de desenvolver os sintomas persistentes. Já os que buscaram atendimento de saúde e fazem uso de medicação contínua apresentam mais chances de sofrer com a Covid longa após 18 meses da infecção primária pelo SARS-CoV-2. Corroborando esses achados, a literatura também aponta que indivíduos com idade superior a 60 anos têm mais chances de desenvolver complicações da Covid-19 a longo prazo^(5)^.

Uma dessas alterações refere-se à qualidade do sono,sendo que diversos estudos afirmam que após a infecção pelo coronavírus os indivíduos passam a apresentar mudanças no padrão do sono. Um estudo realizado em 56 países, onde 3.726 participantes foram acompanhados por sete meses, identificou distúrbios do sono em 78,6% da amostra, 60% referiram insônia, 41% sudorese noturna, 36% despertar durante o sono e 10% apneia^(13)^.

Pessoas idosas que apresentam distúrbios do sono possuem maior predisposição a desenvolver alterações neuropsiquiátricas,como ansiedade, depressão, fadiga, diminuição da concentração, disfunção cognitiva e quadros de parestesia. Logo, ter boa qualidade do sono diminui a ocorrência desses eventos que estão presentes na maioria dos indivíduos com a Covid longa^(14)^.

Em Hubei, província da China, epicentro da pandemia,evidenciou-se que após a fase aguda da doença, 17,7% das pessoas idosas apresentaram alterações no padrão do sono^(15)^. Resultados semelhantes de uma coorte prospectiva conduzida em Hong Kong com essa mesma população encontraram 14,0% com alterações do sono; a insônia e a má qualidade do sono foram os sintomas mais prevalentes^(16)^. Neste estudo, a maior parte dos participantes referiu ter a qualidade do sono como boa e excelente, o que se configurou como um fator protetor para a Covid longa.

Cabe salientar que as pessoas idosas evoluem de forma negativa ao coronavírus, apresentando sequelas e complicações que atingem a sua motricidade e funções cognitivas após a fase aguda da doença, sendo em longo prazo intitulada Covid longa^(17)^.

Outro achado do estudo que vem sendo descrito em outros trabalhos é o uso de medicação contínua como preditor para a Covid longa. Uma pesquisa conduzida em quatro cidades chinesas, com 2.712 indivíduos com sintomas persistentes, identificou que 7,3% da amostra referiram uso de medicação contínua antes de contrair a Covid-19, o que os torna suscetíveis^(18)^. Já outros estudos apontam que, de acordo com a necessidade individual, as alterações da Covid longa são tratadas com uso de medicação diária entre eles: ansiolíticos, medicamentos para depressão, antialérgicos, assim visando minimizar os efeitos pós Covid^(19)^.

Segundo dados de um estudo de coorte, realizado nos Estados Unidos, após um ano da Covid-19 houve um aumento em prescrições medicamentosas, após a fase aguda da doença, entre eles: vitamínicos e medicamentos anticoagulantes^(20)^. Corroborando esses achados, outro estudo mostrou que a utilização de medicamentos para tratar as comorbidades apresentadas em sua maioria por pessoas idosas está associada à Covid longa^(21)^.

Um estudo de coorte conduzido no Irã acompanhou os indivíduos por três meses após o diagnóstico de Covid-19, a fim de identificar os fatores associados à Covid longa. Foram realizadas avaliações clínicas e coleta de exames, os pesquisadores observaram alterações psicológicas e a persistência de sinais e sintomas nos participantes. A amostra do estudo foi composta por 900 pacientes, hospitalizados no início de 2020 e que tiveram alta; destes, 350 realizaram o acompanhamento de saúde após a fase aguda da doença. Pode-se presumir o quão necessária é a busca pelos serviços de saúde para tratar as sequelas após a doença^(22)^.

O cuidado em longo prazo posterior a Covid-19 é essencial para recuperação. Faz-se necessária a comunicação efetiva entre as equipes intra-hospitalar e a assistência de cuidados primários, dispondo-se de informações sobre as condições de saúde dos usuários e a oferta de serviços especializados para essa demanda^(23)^. Sabe-se que os indivíduos que contraíram o coronavírus foram classificados em forma leve, moderada, e a maior parte das pessoas com idade avançada apresentou casos graves; assim, essas requerem acompanhamentos com maior periodicidade^(24)^.

As pessoas idosas possuem uma carga global maior de doenças crônicas, o que a literatura destaca como fator que contribui para a permanência de sintomas e complicações após a fase aguda da doença. Sendo assim, há a necessidade de serviços especializados e profissionais capacitados para realizar os cuidados e atendimentos de saúde de acordo com a especificidade de cada indivíduo. Nesse sentido, é imprescindível a atenção às pessoas idosas a fim de reduzir os danos e melhorar a qualidade de vida^(25)^.

Notoriamente, mesmo com o fim da fase emergencial da pandemia da Covid-19, advêm consigo novos desafios e demandas de cuidado, emergindo as necessidades de modelos de assistência voltados para a pessoa idosa^(13,26)^.

Nesse cenário, as demandas relacionadas à Covid longa estão sendo na maior parte das vezes direcionadas à Atenção Primária à Saúde (APS), a qual realiza tratamentos de baixa complexidade. Em contrapartida, encontram-se diversas adversidades, entre elas, a falta de informações de saúde após a alta,a morosidade em conseguir atendimento pela APS e a ausência de conhecimento acerca dessa condição, o que exige capacitações voltadas à temática da Covid longa e suas implicações na saúde da população^(27)^.

Como limitações do estudo, destaca-se o número de perdas na coleta de dados. Não houve anuência para entrevistas em inúmeros contatos realizados devido ao receio dos participantes em relação a ligações telefônicas mal-intencionadas. Outra limitação refere-se ao viés de recordação, como pode acontecer em entrevistas retrospectivas. No entanto, outro motivo pode ser a falta de compreensão da pessoa idosa sobre a temática, especialmente por se tratar de um formato remoto, além da complexidade do instrumento padronizado, que pode dificultar seu entendimento pelos entrevistados.

Considerando as dificuldades dos participantes idosos em responder ao instrumento de coleta, os autores sugerem, para estudos futuros, investigações que adotem abordagens mais acessíveis e adaptadas a esse público. Isso pode incluir a simplificação da linguagem, o uso de entrevistas presenciais ou assistidas e estratégias que facilitem a compreensão e a expressão das experiências dos idosos. Além disso, recomendam a utilização de recursos apropriados para essa faixa etária, garantindo uma análise direcionada às características da Covid-19 longa neste grupo.

Desta forma, é importante salientar a necessidade de novos estudos relacionados à Covid longa, especialmente com períodos mais longos após o diagnóstico, para que os fatores associados e a persistência dos sinais e sintomas nesse estrato etário sejam embasados com evidências mais consistentes na literatura.

Ao que concerne este aspecto, os achados desta pesquisa contribuem para que os respectivos gestores de saúde possam elaborar e implementar futuras políticas públicas, embasadas na literatura. Assim, podendo realizar ações assertivas voltadas para o atendimento de pessoas idosas diagnosticadas com Covid longa, com norte na recuperação da saúde e prevenção de agravos.

## CONCLUSÃO

Neste estudo, ser tabagista ou ex-tabagista e fazer uso continuamente de medicamentos foram associados a Covid longa. A qualidade do sono autorreferida como boa ou excelente associou-se a Covid longa como fator de proteção.

Considerando a alta carga de comorbidades, o uso frequente de múltiplos medicamentos e a vulnerabilidade dos idosos a doenças infecciosas, é fundamental que os serviços e profissionais de saúde estejam preparados para atender às demandas daqueles que convivem com as sequelas do SARS-CoV-2.

Os achados deste estudo fomentam contribuições para compreensão da Covid longa em pessoas idosas, ampliando o conhecimento científico e ressaltando a necessidade de aprimorar as estratégias voltadas para essa condição.

Espera-se que esses dados impulsionem o progresso no estudo da Covid longa em idosos, oferecendo informações essenciais para a criação de práticas e políticas de saúde que favoreçam a recuperação e melhorem a qualidade de vida dessa população.

## Data Availability

Todo o conjunto de dados que dá suporte aos resultados deste estudo foi publicado no próprio artigo.

# Translation: Prevalencia y factores asociados al Covid prolongado en ancianos del Estado de Paraná^*^

**DOI:** 10.1590/1980-220X-REEUSP-2024-0429es

**Authors:** Isabela Vanessa Tavares Cordeiro Silva, Luiz Hiroshi Inoue, Franciele Aline Machado Brito, Wanessa Cristina Baccon, Giovanna Brichi Pesce, Carla Franciele Höring, Mikhael dos Santos Theodoro, Daiane Cristina Moderno Estevam Inoue, Lorhayne Silveira Dores, Natany Aparecida Batista, Patrícia Bossolani Charlo, Vanessa Denardi Antoniassi Baldissera, Débora Regina de Oliveira Moura, Maria Aparecida Salci, Lígia Carreira

**Affiliations:** 1 Universidade Estadual de Maringá, Maringá, PR, Brasil.; 2 Universidade Unicesumar, Maringá, PR, Brasil.

**Keywords:** Síndrome Post Agudo de COVID-19, Anciano, Calidad del Sueño, Medicamentos de Uso Contínuo, COVID-19

## Abstract

**Objetivo:**

Analizar los factores asociados al Covid prolongado en ancianos 18 meses después de la fase aguda de la enfermedad.

**Método:**

Este estudio transversal y analítico se realizó con ancianos que tuvieron Covid-19 en 2020 y sobrevivieron a la enfermedad en el estado de Paraná, Brasil. Se recogió información sociodemográfica, hábitos de vida, información de salud y tratamiento, así como información sobre signos, síntomas y secuelas de la enfermedad mediante entrevistas telefónicas realizadas 18 meses después de la notificación o el alta hospitalaria. Para los análisis se utilizaron medidas descriptivas, pruebas de asociación y modelos de regresión de Poisson con varianza robusta.

**Resultados:**

De los 345 ancianos que participaron en el estudio, 224 (65%) aún presentaban algún síntoma o secuela de Covid-19 18 meses después de la enfermedad. Ser fumador o exfumador (PR = 2,21; IC 95% = 1,06;3,36), calidad del sueño autoinformada (PR = 0,32; IC 95% = 0,16–0,47) y el uso de medicación continua (PR = 5,24; IC 95%: 2,64–7,85; p < 0,0001) se asociarón con Covid prolongado.

**Conclusión:**

Las tasas de morbilidad y polifarmacia son elevadas en esta población, lo que contribuye a la persistencia de los síntomas. En este contexto, se hace imprescindible alinear los servicios de salud, junto con los profesionales, para atender las necesidades derivadas de las secuelas en las personas mayores.

## INTRODUCCIÓN

La pandemia de COVID-19 es responsable de miles de infecciones y muertes causadas por el agravamiento de la enfermedad. En este contexto, la edad avanzada y la presencia de comorbilidades se consideraron los principales factores de riesgo de complicaciones y muertes^(1)^.

Los datos epidemiológicos de febrero de 2025 apuntan a 777 millones de casos de la enfermedad, con una estimación de 7,1 millones de muertes en todo el mundo^(2)^. En Brasil, en el mismo periodo se registraron 39 millones de infectados, con aproximadamente 715 000 muertes. El estado de Paraná, hasta el 20 de febrero de 2025, presentaba poco más de 3 millones de casos y 47 mil muertes, de las cuales 30 287 correspondían a personas de 60 años o más^(3,4)^.

Existe consenso en la literatura científica en que las personas mayores son más susceptibles de desarrollar las formas más graves de COVID-19. Uno de los factores que contribuye a esta susceptibilidad es la inmunosenescencia, un proceso que consiste en el deterioro de las funciones del sistema inmunitario, sumado a la alta carga de enfermedades preexistentes en este estrato de la población, y que puede contribuir a la persistencia de los síntomas después de la fase aguda, una condición conocida como COVID prolongado^(5)^.

El COVID prolongado se caracteriza por la persistencia de signos y síntomas durante más de tres meses después de la fase aguda de la enfermedad. La Organización Mundial de la Salud (OMS) estima que entre el 10 y el 20% de los pacientes que han contraído COVID-19 presentarán secuelas a medio y largo plazo. Sin embargo, aún no existe una base de datos con información sobre las personas afectadas por el síndrome pos-COVID-19. Esta condición ha alertado a las autoridades sanitarias y ha aumentado la demanda de servicios especializados^(6)^. Además, el COVID prolongado puede afectar negativamente a las actividades básicas de la vida diaria (ABVD) de las personas afectadas^(7,8)^.

Ante la complejidad del COVID prolongado y el impacto que tiene en la salud física y emocional, especialmente en la población de edad avanzada, aún se necesitan estudios que aborden la prevalencia y los factores asociados a esta condición^(7)^, ya que esta nueva demanda de cuidados y búsqueda de atención en los servicios de salud configura la necesidad de un nuevo formato de asistencia, con servicios especializados orientados a la recuperación y la reducción de las secuelas post-COVID-19, así como nuevas competencias por parte del equipo multidisciplinario para manejar esta condición, ya que aún no existen directrices claras sobre el tratamiento del COVID prolongado^(9)^.

Así, considerando la vulnerabilidad de las personas mayores frente al Covid largo y la falta de evidencia sobre el síndrome post-Covid-19, este estudio se propuso analizar los factores asociados a esta condición después de 18 meses de la infección primaria por SARS-CoV-2.

## MÉTODO

### Diseño del Estudio

Se trata de una encuesta observacional transversal, individualizada y analítica, integrada en una cohorte titulada “Seguimiento longitudinal de adultos y personas mayores que han sido dados de alta del hospital por COVID-19” (Cohorte COVID-19 Paraná/UEM)^(10)^.

### Lugar

El lugar del estudio fue el estado de Paraná, situado en la región sur de Brasil. En 2021, la población estimada del estado era de aproximadamente 11,6 millones de habitantes, con 1,9 millones de personas mayores^(11)^ y un Índice de Desarrollo Humano (IDH) de 0,769^(12)^.

### Población

La población del estudio estaba compuesta por personas mayores (60 años o más) que tuvieron Covid-19 en el año 2020 y sobrevivieron a la enfermedad 18 meses después de la notificación o el alta hospitalaria.

### Criterios de Selección

Se utilizaron dos bases de datos para la selección de los participantes: el Sistema de Información de Vigilancia Epidemiológica de la Gripe (SIVEP-Gripe), donde se registran los casos que requieren hospitalización en salas y unidades de terapia intensiva (UTI), y las muertes, independientemente de la hospitalización, y el Sistema Estatal Notifica Covid-19 (Notifica Covid-19), una base de datos elaborada e implementada por el gobierno del estado de Paraná, con el fin de registrar los casos, hospitalizaciones y muertes del propio estado.

A partir de un análisis previo de la integridad de las bases de datos que contienen la información sensible de los participantes, se utilizaron cuatro variables para el proceso de selección: nombre, fecha de nacimiento, sexo y nombre de la madre. Tras el tratamiento de los datos, el SIVEP-Gripe se utilizó exclusivamente para los casos que requirieron hospitalización, mientras que Notifica Covid-19 se empleó para los casos leves, es decir, aquellos con resultado positivo para la enfermedad que no necesitaron hospitalización.

Como criterios de selección se consideraron las personas de 60 años o más, residentes y notificadas en Paraná, con diagnóstico confirmado de Covid-19 mediante la prueba RT-PCR, que habían salido del tratamiento en enfermería o UCI y de la atención ambulatoria (no hospitalizada) en el periodo de marzo a diciembre de 2020, con número de teléfono fijo o celular correctamente rellenado. Se excluyó del estudio a los pacientes que fallecieron.

### Muestra

Inicialmente, se seleccionaron 1500 participantes a partir de bases de datos utilizando un muestreo aleatorio probabilístico estratificado proporcional según el mes de notificación o alta hospitalaria (de marzo a diciembre de 2020) y la macrorregión sanitaria de residencia (Oeste, Este, Norte y Noroeste), según el protocolo descrito por Salci et al.^(10)^.

En el primer contacto telefónico, realizado 12 meses después de la notificación o el alta, 900 personas mayores aceptaron participar en la investigación. En un segundo contacto, 18 meses después de la notificación o el alta hospitalaria, 345 personas mayores respondieron efectivamente a la investigación (Figura 1).

**Figura 1:** Proceso de selección de la muestra a partir de las bases de datos.

Fuente: Elaborado por los autores.

### Recopilación de Datos

Los datos se recopilaron mediante entrevistas telefónicas realizadas 18 meses después de la notificación (para los casos leves) o del alta hospitalaria (para los casos moderados y graves), en el periodo comprendido entre noviembre de 2021 y septiembre de 2022. Se invitó a las personas a participar en el estudio por teléfono a través de un equipo de enfermeras, estudiantes de grado y posgrado del área de la salud, que había sido formado y capacitado previamente durante dos meses. Se aclararon los objetivos de la investigación y se obtuvo el consentimiento verbal tras la lectura del Término de Consentimiento Libre y Esclarecido (TCLE). En los casos en que el participante no podía comunicarse en el momento del contacto, se permitió que el cuidador o familiar responsable respondiera a las preguntas del formulario. Las entrevistas tuvieron una duración media de 30 minutos.

Como instrumento de recopilación de datos, se utilizó un formulario de investigación estructurado que incluía preguntas sociodemográficas, sobre hábitos de vida, salud y tratamiento de la COVID-19, y abordaba los signos y síntomas de la enfermedad. El formulario fue elaborado y validado por los investigadores de la cohorte^(10)^.

### Variables del Estudio

Se definió como variable dependiente el Covid prolongado (sí y no), caracterizado en este estudio por la presencia de al menos uno de los signos o síntomas autoinformados (enumerados a continuación), persistentes después de 18 meses desde la notificación o el alta hospitalaria.

Los signos y síntomas evaluados se agruparon según los siguientes sistemas del cuerpo humano: neurológico (cefalea, dolor ocular, alteración de la visión, alteración del olfato, alteración del gusto, alteración del habla, alteración de la audición, zumbido en los oídos, mareos, pérdida de coordinación motora, pérdida/disminución de la memoria, hormigueo/entumecimiento, desmayos, fiebre, ansiedad y depresión); respiratorio (congestión nasal, dolor de garganta, voz ronca, tos, producción de catarro, dolor torácico y disnea); digestivo (alteración de las heces, alteración del apetito, náuseas, cólicos/dolor abdominal y vómitos); endocrino (caída del cabello y sudoración); cutáneo (manchas en el cuerpo y prurito en el cuerpo); musculoesquelético (problemas en los músculos/articulaciones, cansancio/fatiga); y cardiovascular (edema).

Las variables independientes se definieron en cuatro ámbitos: (1) sociodemográficas; (2) hábitos de vida; (3) condiciones de salud; y (4) tratamiento de la COVID-19.

1. Sociodemográficas: sexo (masculino y femenino), edad (60 a 74 años y 75 años o más), raza/color (blanco/amarillo y negro/indígena), años de estudio (hasta 8 y 8 o más), si tiene pareja (a) (no y sí), si vive solo(a) (no y sí) e ingresos familiares (hasta dos y más de dos salarios mínimos);
2. Hábitos de vida: consumo de alcohol (no y sí), fumador o exfumador (no y sí), práctica de actividad física (no y sí) y calidad del sueño autoinformada (muy mala/mala/regular y buena/excelente);
3. Condiciones de salud: padecer morbilidad (no y sí), tomar medicamentos de uso continuo (no y sí), haber buscado atención médica en los últimos 18 meses (no y sí), necesitar ayuda/cuidador (no y sí) y presentar síntomas en la fase aguda de la COVID-19 (no y sí);
4. Tratamiento de la COVID-19 en la fase aguda: lugar (consultorio, enfermería y unidad de terapia intensiva), uso de asistencia respiratoria (no y sí) y si utilizó el Sistema Único de Salud (SUS) en alguna fase del tratamiento (no y sí).

Las preguntas que los participantes optaron por no responder se clasificaron como “no informadas”.

### Análisis y Tratamiento de los Datos

Se realizaron análisis descriptivos de los resultados, así como asociaciones de las variables independientes (sociode-mográficas, hábitos de vida, condiciones de salud y tratamiento de la COVID-19) con el resultado (presencia de COVID prolongada después de 18 meses) mediante la prueba de chi cuadrado de Pearson o la prueba exacta de Fisher, según fuera necesario para cada tipo de variable, con un nivel de significación del 5%.

Para identificar los factores asociados a los signos y síntomas persistentes, se aplicaron modelos de regresión de Poisson con varianza robusta. Las asociaciones se estimaron mediante la razón de prevalencias (RP), adoptando un intervalo de confianza del 95% como medida de precisión.

El modelo final se ajustó considerando p < 0,20 en el análisis bivariado y utilizando el *método stepwise both* para la selección de las variables. La prueba de hipótesis aplicada para verificar la significación de los coeficientes estimados fue la prueba de Wald y la adecuación del modelo se evaluó mediante el análisis de los residuos de desviación y cuartílico aleatorios, y del factor de inflación de la varianza (VIF) de las variables independientes. Además, se calculó el pseudo coeficiente de determinación R^2^ de Nagelkerke para verificar la calidad de los ajustes.

Los datos recopilados en las entrevistas se organizaron en hojas de cálculo y se analizaron mediante el *software* R versión 4.5.0.

### Aspectos Éticos

La investigación fue aprobada por el Comité Permanente de Ética en Investigación con Seres Humanos de la Universidad Estatal de Maringá/PR, con el dictamen n.º 4.165.272/2020 y CAAE: 34787020.0.0000.0104 el 21 de julio de 2020. Además, para los datos obtenidos de Notifica Covid, recibió la aprobación de la Secretaría de Salud del Estado de Paraná, a través del Hospital del Trabajador, obteniendo el dictamen n.º 4.214.589/2020 y CAAE: 34787020.0.3001.5225, el 15 de agosto de 2020.

## RESULTADOS

Participaron en el estudio 345 personas mayores, con una edad media de 68 años, una mediana de 66 años y una desviación estándar de 6,6 años.

La tabla 1 contiene las características de la muestra según las variables sociodemográficas, los hábitos de vida, la salud y el tratamiento de la Covid-19.

**Tabla 1:** Características sociodemográficas, hábitos de vida, salud y tratamiento de la COVID-19 en personas mayores 18 meses después de la fase aguda de la enfermedad en el estado de Paraná, Brasil, 2021-2022 (n = 345) – Maringá, PR, Brasil, 2025.

Características	Categoría	n	%
**Sociodemográficas**			
Sexo	Masculino	172	49,9
	Femenino	173	50,1
Rango de edad (años)	60 a 74 años	282	81,7
	75 o más	63	18,3
Raza/Color	Blanca/Amarilla	173	50,1
	Negra/Indígena	63	18,3
	No informado	109	31,6
Años de estudio	Hasta 8	118	34,2
	Más de 8	94	27,2
	No informado	133	38,6
Tiene pareja	No	83	24,1
	Sí	196	56,8
	No informado	66	19,1
Vive solo	No	252	73,0
	Sí	51	14,8
	No informado	42	12,2
Ingresos familiares^*^	≤2 salarios mínimos	60	17,4
	>2 s.m.	84	24,3
	No informado	201	58,3
**Hábitos de vida**
Consumo de bebidas alcohólicas	No	111	32,2
	Sí	159	46,1
	No informado	75	21,7
Fumador o exfumador	No	148	42,9
	Sí	87	25,2
	No informado	110	31,9
Práctica de actividad física	No	69	20,0
	Sí	175	50,7
	No informado	101	29,3
Calidad del sueño autoinformada	Mala/regular/pésima	63	18,3
	Buena/excelente	242	70,1
	No informado	40	11,6
**Salud**			
Morbidez	No	123	35,7
	Sí	222	64,3
Medicamentos de uso continuo	No	165	47,8
	Sí	88	25,5
	No informado	92	26,7
Búsqueda de atención sanitaria	No	131	38,0
	Sí	137	39,7
	No informado	77	22,3
Necesidad de ayuda/cuidador(a)	No	141	40,9
	Sí	119	34,5
	No informado	85	24,6
Síntomas en la fase aguda	No	110	32,0
	Sí	235	68,0
**Tratamiento**
Lugar del tratamiento	Ambulatorio	148	42,9
	Enfermería	102	29,6
	UTI	95	27,5
Uso de soporte ventilatorio	No	172	49,9
	Sí	130	37,7
	No informado	43	12,5
Tratamiento en el SUS	No	2	0,6
	Sí	175	50,7
	No informado	168	48,7

*Se utilizó el salario mínimo (s.m.) de 1210,00 reales, que corresponde al valor vigente en el momento del inicio de la investigación; SUS: Sistema Único de Salud; UTI: Unidad de Terapia Intensiva.

En cuanto a la información sociodemográfica, la mayoría de las personas mayores tenían entre 60 y 74 años, eran mujeres, se identificaban como blancas/amarillas, tenían hasta ocho años de estudios, vivían con su pareja, no residían solas y tenían ingresos familiares superiores a dos salarios mínimos (Tabla 1).

En cuanto a los hábitos de vida, la mayoría de los participantes consumen bebidas alcohólicas, no fuman, practican actividad física y consideran que la calidad de su sueño es buena/excelente (Tabla 1).

En cuanto a las condiciones de salud, la mayoría tiene una o más morbilidades, toma medicación de forma continua, ha buscado atención médica y ha necesitado ayuda o incluso un cuidador debido a las complicaciones causadas por la COVID-19.

En cuanto al tratamiento de la enfermedad en la fase aguda, la muestra estaba compuesta principalmente por personas atendidas en ambulatorios, que no necesitaron asistencia respiratoria y que utilizaron los servicios del SUS (Sistema Único de Salud) en alguna fase del tratamiento.

Entre los más prevalentes, destacan la pérdida/disminución de la memoria, la ansiedad, la depresión, la disnea, la caída del cabello, los problemas musculares/articulares y la sensación de cansancio/fatiga (Tabla 2).

**Tabla 2:** Signos y síntomas según los sistemas del cuerpo humano de personas mayores 18 meses después de la fase aguda de la COVID-19 en el estado de Paraná, Brasil, 2021-2022 (n = 224) – Maringá, PR, Brasil, 2025.

Signos y síntomas	n	%
**Neurológico**	**167**	**74,6**
Cefalea	29	12,9
Dolor ocular	27	12,1
Alteración de la visión	38	17,0
Alteración del olfato	31	13,8
Alteración del gusto	29	12,9
Alteración en el habla	8	3,6
Alteración en la audición	40	17,9
Zumbido en los oídos	34	15,2
Mareos	51	22,8
Pérdida de coordinación motora	38	17,0
Pérdida/disminución de la memoria	123	54,9
Hormigueo o entumecimiento en el cuerpo	44	19,6
Desmayo	4	1,8
Fiebre	2	0,9
Ansiedad	66	29,5
Depresión	61	27,2
**Respiratorio**	**107**	**47,8**
Coriza	37	16,5
Dolor de garganta	15	6,7
Voz ronca	26	11,6
Tos	46	20,5
Producción de flemas	24	10,7
Dolor torácico	17	7,6
Disnea	60	26,8
**Digestivo**	**56**	**25,0**
Alteración en las heces	18	8,0
Alteración en el apetito	37	16,5
Náuseas	15	6,7
Cólicos/dolor abdominal	12	5,4
Vómitos	2	0,9
**Endocrino**	**68**	**30,4**
Caída del cabello	54	24,1
Sudoración	19	8,5
**Cutáneo**	**23**	**10,3**
Manchas por el cuerpo	5	2,2
Prurito corporal	22	9,8
**Musculoesquelético**	**137**	**61,2**
Problemas en los músculos/articulaciones	57	25,4
Cansancio/fatiga	123	54,9
**Cardiovascular**	**17**	**7,6**
Edema	17	7,6

En el modelo bruto, las variables asociadas al Covid prolongado (p < 0,05) fueron los años de estudio, la calidad del sueño autoinformada, la morbilidad, la medicación de uso continuo, la búsqueda de atención médica, el número de síntomas en la fase aguda de la enfermedad y la hospitalización en la UCI (Tabla 3).

**Tabla 3:** Modelo bruto para las asociaciones entre las características sociodemográficas, los hábitos de vida, la salud, el tratamiento y el Covid prolongado en personas mayores 18 meses después de la fase aguda de la enfermedad en el estado de Paraná, Brasil, 2021-2022 (n = 345) – Maringá, PR, Brasil, 2025.

Variables	Categorías	Covid prolongado (n = 224; 65%)		RP (IC 95%)
		n (%)	valor p	
**Sociodemográficas**				
Sexo	Masculino	104 (60,4)	0,1054	Ref.
	Femenino	120 (69,3)		0,88 (0,75–1,02)
Rango de edad (años)	60 a 74 años	187 (66,3)	0,3202	Ref.
	75 o más	37 (58,7)		1,13 (0,91–1,42)
Raza/Color	Blanca/Amarilla	121 (69,9)	0,7471	Ref.
	Negra/Indígena	42 (66,6)		1,06 (0,86–1,29)
Años de estudio	Hasta 8	87 (73,7)	0,0600	Ref.
	Más de 8	57 (60,6)		1,20 (0,99–1,46)
Tiene pareja	No	55 (66,2)	0,6473	Ref.
	Sí	137 (69,8)		0,94 (0,79–1,13)
Vive solo	No	170 (67,4)	0,9988	Ref.
	Sí	35 (68,6)		0,99 (0,80–1,21)
Renta familiar	≤2 s.m.	45 (75)	0,7754	Ref.
	>2 s.m.	60 (71,4)		1,07 (0,88–1,30)
**Hábitos de vida**				
Consumo de bebidas alcohólicas	No	73 (65,7)	0,4280	Ref.
	Sí	113 (71,0)		0,93 (0,79–1,10)
Fumador o exfumador	No	98 (66,2)	0,1097	Ref.
	Sí	67 (77,0)		0,87 (0,74–1,02)
Práctica de actividad física	No	53 (76,8)	0,1711	Ref.
	Sí	117 (66,8)		1,14 (0,97–1,35)
Calidad del sueño autoinformada	Mala/regular/pésima	58 (92,0)	**<0,0001**	Ref.
	Buena/excelente	144 (59,5)		1,54 (1,36–1,75)
**Salud**				
Morbilidad	No	69 (56,0)	**0,0147**	Ref.
	Sí	155 (69,8)		0,81 (0,68–0,97)
Medicamentos de uso continuo	No	104 (63,0)	**0,0105**	Ref.
	Sí	70 (79,5)		0,80 (0,68–0,93)
Búsqueda de atención médica	No	68 (51,9)	**<0,0001**	Ref.
	Sí	118 (86,1)		0,61 (0,51–0,73)
Necesidad de ayuda/cuidador(a)	No	99 (70,2)	0,5024	Ref.
	Sí	78 (65,5)		1,08 (0,91–1,28)
Síntomas en la fase aguda	No	66 (60,0)	0,1722	Ref.
	Sí	158 (67,5)		0,89 (0,74–1,06)
**Tratamiento**				
Lugar de tratamiento	Ambulatorio	93 (62,8)	**0,0241**	Ref.
	Enfermería	59 (57,8)		1,09 (0,89–1,34)
	UTI	72 (75,7)		0,83 (0,71–0,99)
Uso de soporte ventilatorio	No	111 (64,5)	0,5542	Ref.
	Sí	89 (68,4)		0,95 (0,81–1,11)
Tratamiento en el SUS	No	1 (50,0)	0,5183^b^	Ref.
	Sí	122 (69,7)		0,71 (0,18–2,86)

UTI: Unidad de Terapia Intensiva; SUS: Sistema Único de Salud; ^a^Prueba de chi cuadrado de Pearson;

^b^Prueba exacta de Fisher; IC 95%: intervalo de confianza del 95%; RPb: razón de prevalencias brutas; Ref.: categoría de referencia; Los valores resaltados indican p < 0,05. s.m.: salarios mínimos.

La Tabla 4 presenta el modelo final ajustado para la prevalencia del Covid prolongado en personas mayores según las covariables del estudio. Se observó que las personas mayores fumadoras o exfumadoras presentaban una prevalencia de Covid prolongado 2,21 veces mayor en comparación con las no fumadoras. La calidad del sueño autodefinida como buena o excelente presentó una prevalencia de 0,32 en comparación con aquellos que la definieron como pésima, mala o regular. Las personas mayores que toman medicación de forma continua presentaron una prevalencia de COVID prolongado 5,24 veces mayor en comparación con las que no la toman. Las variables “búsqueda de atención médica en los últimos 18 meses” y “presencia de síntomas en la fase aguda de la enfermedad” no fueron significativas, pero se mantuvieron en el modelo debido a los supuestos y las pruebas de adecuación del ajuste.

**Tabla 4:** Modelo final ajustado para las asociaciones de las variables de hábitos de vida, salud y tratamiento de la COVID-19 en personas mayores 18 meses después de la fase aguda en el estado de Paraná, Brasil, 2021-2022 (n = 345) – Maringá, PR, Brasil, 2025.

Variables	Categorías	RP (IC 95%)	Valor p
Intercepto	–	–	**0,0305**
Fumador o exfumador	Sí	2,21 (1,06;3,36)	**0,0270**
Calidad del sueño autoinformada	Buena/excelente	0,32 (0,16;0,47)	**0,0032**
Medicamentos de uso continuo	Sí	5,24 (2,64;7,85)	**<0,0001**
Búsqueda de atención médica	Sí	1,73 (0,70;2,76)	0,1516
Síntomas en la fase aguda	Sí	0,23 (0,05;1,10)	0,0706

IC 95%: intervalo de confianza del 95%; RP: razón de prevalencias; los valores resaltados indican p < 0,05.

La información sobre las variables de tratamiento en el SUS no se incluyó en el modelo final, ya que no contenía muestras suficientes para los ajustes. El uso de soporte ventilatorio presentó colinealidad con el lugar de tratamiento y la morbilidad, colinealidad con el uso de medicación continua. Los resultados de las pruebas de residuos *deviantes* presentaron p = 0,4780 y los cuartiles aleatorios p = 0,5477, y el resultado del pseudo R^2^ ajustado fue del 92,2%, el mejor entre los modelos evaluados.

## DISCUSIÓN

Los resultados de este análisis demostraron que las personas mayores, al ser más vulnerables a la COVID-19, se volvieron más susceptibles a la COVID prolongada. Además, evidenciaron que aquellos que califican la calidad del sueño como buena y excelente tienen menos probabilidades de desarrollar síntomas persistentes. Por otro lado, aquellos que buscaron atención médica y toman medicación de forma continua tienen más probabilidades de sufrir Covid prolongado después de 18 meses de la infección primaria por SARS-CoV-2. Corroborando estos hallazgos, la literatura también señala que las personas mayores de 60 años tienen más probabilidades de desarrollar complicaciones de Covid-19 a largo plazo^(5)^.

Una de estas alteraciones se refiere a la calidad del sueño, y varios estudios afirman que, tras la infección por coronavirus, las personas comienzan a presentar cambios en sus patrones de sueño. Un estudio realizado en 56 países, en el que se siguió a 3726 participantes durante siete meses, identificó trastornos del sueño en el 78,6% de la muestra, el 60% refirió insomnio, el 41% sudoración nocturna, el 36% despertares durante el sueño y el 10% apnea^(13)^.

Las personas mayores que presentan trastornos del sueño tienen una mayor predisposición a desarrollar alteraciones neuropsiquiátricas, como ansiedad, depresión, fatiga, disminución de la concentración, disfunción cognitiva y cuadros de parestesia. Por lo tanto, tener una buena calidad de sueño disminuye la ocurrencia de estos eventos que están presentes en la mayoría de los individuos con Covid prolongado^(14)^

En Hubei, provincia de China, epicentro de la pandemia, se evidenció que, tras la fase aguda de la enfermedad, el 17,7% de las personas mayores presentaban alteraciones en el patrón de sueño^(15)^. Resultados similares de una cohorte prospectiva realizada en Hong Kong con esta misma población encontraron que el 14,0% presentaba alteraciones del sueño; el insomnio y la mala calidad del sueño fueron los síntomas más prevalentes^(16)^. En este estudio, la mayoría de los participantes referían tener una calidad del sueño buena o excelente, lo que se configuró como un factor protector para el Covid prolongado.

Cabe destacar que las personas mayores evolucionan de forma negativa al coronavirus, presentando secuelas y complicaciones que afectan a su motricidad y funciones cognitivas tras la fase aguda de la enfermedad, lo que a largo plazo se denomina Covid prolongado^(17)^.

Otro hallazgo del estudio que se ha descrito en otros trabajos es el uso de medicación continua como predictor del Covid largo. Una investigación realizada en cuatro ciudades chinas, con 2712 individuos con síntomas persistentes, identificó que el 7,3% de la muestra refería el uso de medicación continua antes de contraer el Covid-19, lo que los hace susceptibles^(18)^. Otros estudios señalan que, según las necesidades individuales, los cambios del Covid largo se tratan con el uso de medicación diaria, entre ellos: ansiolíticos, medicamentos para la depresión, antialérgicos, con el fin de minimizar los efectos post-Covid^(19)^.

Según los datos de un estudio de cohorte realizado en Estados Unidos, un año después de la COVID-19 se produjo un aumento de las recetas médicas, tras la fase aguda de la enfermedad, entre las que se encontraban: vitaminas y anticoagulantes^(20)^. Corroborando estos hallazgos, otro estudio demostró que el uso de medicamentos para tratar las comorbilidades que presentan en su mayoría las personas mayores está asociado a la COVID prolongada^(21)^.

Un estudio de cohorte realizado en Irán siguió a los individuos durante tres meses tras el diagnóstico de COVID-19, con el fin de identificar los factores asociados al COVID prolongado. Se realizaron evaluaciones clínicas y se recogieron pruebas, y los investigadores observaron cambios psicológicos y la persistencia de signos y síntomas en los participantes. La muestra del estudio estuvo compuesta por 900 pacientes, hospitalizados a principios de 2020 y que fueron dados de alta; de ellos, 350 realizaron un seguimiento de salud después de la fase aguda de la enfermedad. Se puede suponer lo necesario que es buscar servicios de salud para tratar las secuelas después de la enfermedad^(22)^.

La atención a largo plazo después de la COVID-19 es esencial para la recuperación. Es necesaria una comunicación eficaz entre los equipos intrahospitalarios y la asistencia de atención primaria, disponiendo de información sobre las condiciones de salud de los usuarios y la oferta de servicios especializados para esta demanda^(23)^. Se sabe que las personas que contrajeron el coronavirus se clasificaron en forma leve, moderada, y la mayoría de las personas de edad avanzada presentaron casos graves; por lo tanto, estas requieren un seguimiento más frecuente^(24)^.

Las personas mayores tienen una mayor carga global de enfermedades crónicas, lo que la literatura destaca como un factor que contribuye a la persistencia de los síntomas y las complicaciones después de la fase aguda de la enfermedad. Por lo tanto, se necesitan servicios especializados y profesionales capacitados para realizar la atención y los cuidados de salud de acuerdo con las especificidades de cada individuo. En este sentido, es imprescindible prestar atención a las personas mayores con el fin de reducir los daños y mejorar su calidad de vida^(25)^.

Es evidente que, incluso con el fin de la fase de emergencia de la pandemia de COVID-19, surgen nuevos retos y demandas de atención, lo que hace que se plantee la necesidad de modelos de asistencia orientados a las personas mayores^(13,26)^.

En este contexto, las demandas relacionadas con la COVID prolongada se dirigen en su mayor parte a la atención primaria de salud (APS), que realiza tratamientos de baja complejidad. Por otro lado, existen diversas adversidades, entre ellas, la falta de información sanitaria tras el alta, la lentitud en conseguir atención por parte de la APS y la falta de conocimiento sobre esta afección, lo que exige una formación centrada en el tema del Covid largo y sus implicaciones para la salud de la población^(27)^.

Entre las limitaciones del estudio, cabe destacar el número de pérdidas en la recopilación de datos. No se obtuvo el consentimiento para realizar entrevistas en numerosos contactos debido al temor de los participantes a las llamadas telefónicas malintencionadas. Otra limitación se refiere al sesgo de recuerdo, como puede ocurrir en las entrevistas retrospectivas. Sin embargo, otra razón puede ser la falta de comprensión del tema por parte de las personas mayores, especialmente al tratarse de un formato remoto, además de la complejidad del instrumento estandarizado, que puede dificultar su comprensión por parte de los entrevistados.

Teniendo en cuenta las dificultades de los participantes de edad avanzada para responder al instrumento de recopilación, los autores sugieren, para futuros estudios, investigaciones que adopten enfoques más accesibles y adaptados a este público. Esto puede incluir la simplificación del lenguaje, el uso de entrevistas presenciales o asistidas y estrategias que faciliten la comprensión y la expresión de las experiencias de las personas mayores. Además, recomiendan el uso de recursos apropiados para este grupo de edad, garantizando un análisis dirigido a las características del Covid-19 prolongado en este grupo.

Por lo tanto, es importante destacar la necesidad de nuevos estudios relacionados con el Covid prolongado, especialmente con períodos más largos después del diagnóstico, para que los factores asociados y la persistencia de los signos y síntomas en este estrato etario se basen en evidencias más consistentes en la literatura.

En lo que respecta a este aspecto, los hallazgos de esta investigación contribuyen a que los respectivos gestores de salud puedan elaborar e implementar futuras políticas públicas, basadas en la literatura. De este modo, se pueden llevar a cabo acciones asertivas dirigidas a la atención de personas mayores diagnosticadas con Covid prolongado, con el objetivo de recuperar la salud y prevenir agravamientos.

## CONCLUSIÓN

Por los resultados de este estudio, ser fumador o exfumador y hacer uso continuo de medicamentos se asoció con el Covid largo. La calidad del sueño autodefinida como buena o excelente se asoció con el Covid largo como factor de protección.

Teniendo en cuenta la elevada carga de comorbilidades, el uso frecuente de múltiples medicamentos y la vulnerabilidad de las personas mayores a las enfermedades infecciosas, es fundamental que los servicios y los profesionales de la salud estén preparados para atender las demandas de quienes conviven con las secuelas del SARS-CoV-2.

Los hallazgos de este estudio fomentan contribuciones para la comprensión del Covid largo en personas mayores, ampliando el conocimiento científico y destacando la necesidad de mejorar las estrategias dirigidas a esta condición.

Se espera que estos datos impulsen el progreso en el estudio del Covid largo en personas mayores, ofreciendo información esencial para la creación de prácticas y políticas de salud que favorezcan la recuperación y mejoren la calidad de vida de esta población.

## Data Availability

Todo el conjunto de datos que apoya los resultados de este estudio fue publicado en el propio artículo.
